# Neonatal Kabuki syndrome caused by *KMT2D* mutation: A case report

**DOI:** 10.1097/MD.0000000000036681

**Published:** 2023-12-15

**Authors:** Zhang Li, Zou Ning

**Affiliations:** a The Second Affiliated Hospital of Dalian Medical University, Dalian City, Liaoning Province, China.

**Keywords:** exon sequencing, high, Kabuki syndrome, *KMT2D*, throughput whole

## Abstract

**Background:**

Kabuki syndrome (KS) is an autosomal dominant inherited syndrome that involves multiple organs and systems. Gene mutation is the main cause of KS. The reported mutations in X-linked histone H3 lysine 4 methylase (KMT2D) and KDM6A genes are 2 relatively clear pathogenic pathways. In this paper, we report a case of KS with neonatal hypoglycemia and special features caused by KMT2D gene mutation confirmed by whole exome sequencing, it enriched the clinical phenotype spectrum and gene mutation spectrum of KS, which helps to improve the understanding of the disease.

**Case report:**

Through whole exome sequencing, we performed gene diagnosis of a newborn child with special facial features and multiple malformations, which revealed heterozygous mutation of NM_003482.3:c.755dupA(p.His252Glnfs*21) in *KMT2D* gene. It is consistent with the pathogenesis of KS, an autosomal dominat genetic disease caused by *KMT2D* gene mutation. This pathogenic mutation has not been prebiously reported.

**Discussion:**

KS has strong clinical characteristics and biological heterogeneity. Genetic diagnosis can help identify mutant gene types. However, the relationship between genotype and phenotype has not been fully clarified. The molecular etiological mechanism still needs to be further explored and elucidated.

## 1. Introduction

Kabuki syndrome (KS) is an autosomal dominant inherited disease involving multiple organs and systems. It was first reported in 1981 by Japanese scholars Niikawa and Kuroki. The major clinical manifestations include mental retardation, unique facial structure (long eye crack, large ears, and sunken nose tip), skeletal abnormalities, unusual skin lines, and growth retardation. The incidence of this condition is 1 in every 32,000 people based on Japanese studies.^[1,2]^ Further, KS is primarily caused by genetic mutations. *KMT2D* and *KDM6A* are 2 common causative genes. Type 1 KS, which is caused by the *KMT2D* mutation, is frequently observed^[3,4]^ KS involves multiple systems and organs. The severity of symptoms in each organ is different, and the association and mechanism between genotype and clinical phenotype remains unclear. More data on the genetic mutation spectrum and clinical phenotype of patients with KS are collected and analyzed. Facial abnormalities in the neonatal period are less significant. Hence, KS is challenging to detect within a short period of growth and dysplasia, and it is even more difficult to diagnose. At present, there are only a few reports on confirmed cases of KS in the neonatal period, and neonatal KS is commonly diagnosed^[5]^ based on the presence of congenital developmental malformation. The genetic diagnosis of neonatal KS in the neonatal period is based on clinical manifestations and the type of gene mutation (*KMT2D* mutation).

## 2. Case report

### 2.1. Target child

A male neonate was delivered via cesarean section at 38 weeks of gestation. Her mother (G2P1) had an unexplained spontaneous abortion during her first pregnancy. The patient birth weight was 2630 g, and his Apgar score was 10. Two hours after birth, he was admitted to the neonatal department due to low blood glucose levels and shortness of breath for 1 hour. The parents were not closely related and denied familial genetic history.

### 2.2. Physical examination

**Figure 1.**
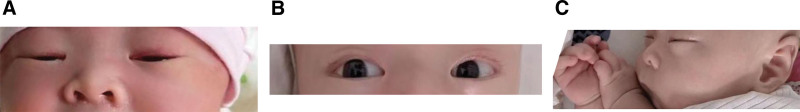
The patient with Kabuki syndrome presented with long eye crack, small eyeball in the left eye, short nose column, large ears, neck webbing, and abnormal palmprint.

Physical examination showed the following results: body temperature: 36.3ºC, heart rate: 152 beats/min, respiratory rate: 58 times/min, blood pressure: 72/36 mm Hg, and percutaneous oxygen saturation without oxygen treatment: of 85%. The patient was reactive and had low-pitched crying and shortness of breath. Face and body cadres can be seen in multiple ecchymoses. The anterior fontanel had a normal tension, size (2.0 × 2.0 cm), and cranial suture separation and patent posterior fontanel. His 2 ears were large, low in position, and spoon-shaped. Further, his eye fissure was long, and the left eyeball was small (Fig. 1A and B). His pupil size was approximately 3 mm, and the light reflex was slow. His right eyeball was normal. The pupil size was approximately 2 mm, and the pupil was sensitive to light reflection. The patient neck was soft and webbed. Double lung auscultation breathing sounds were thick, and no dry and wet breath sounds were heard. The patient had a strong heartbeat and full rhythm, without pathological murmur. His abdomen was soft, without gastrointestinal pattern and peristaltic wave. Moreover, he had small liver and spleen and normal bowel sounds. The patient umbilical cord was detached, and purulent secretions were not observed. The patient umbilical wheel was not red, and his limbs could be moved freely and were cool. Common palm were not observed (Fig. 1C). The patient tested negative for scarf sign and neck pull sign. His limbs had weak muscle tone, and the capillary refill time was 3 seconds. The patient penis was short, with bilateral testis descending to the scrotum. The initial sucking and feeding reflex, but not the holding and embrace reflex, was normal.

### 2.3. Laboratory test results

The blood gas analysis results were as follows: pH, 7.30; PCO2, 45.9 mm Hg; PO2, 62.7 mm Hg; cHCO3act level, 26.8 mmol/L; cBE (vv), −4.1; GLU level, 2.1 mmol/L; lactate level, 3.21 mmol/L; Ca^2+^ level,1.37 mmol/L; Na^+^ level, 138 mmol/L; K^+^ level, 4.3 mmol/L; and Cl level, 113 mmol/L.

The routine blood test results were as follows: + CRP; white blood cell count, 18.07109/L; NEUT%, 68.9%; HIGB level, 201 g/L; platelet count, 120.00 × 109/L; C-reactive protein level, <0.80 mg/L; and procalcitonin level, 3.04 mg/mL.

The liver biochemistry results were as follows: alanine transaminase level, 11 U/L; aspartate aminotransferase level, 39 U/L; albumin level, 29.4 g/L; glutamic acid level, 1.97 mmol/L; hydroxybutyrate dehydrogenase level, 375 U/L; creatine kinase level, 575 U/L; creatine kinase-myocardial band level, 56 U/L; serum insulin level, 41.71 pmol/L; serum C peptide level, 933.14 pmol/L; adrenocorticotropic hormone level, 24.40 pg/mL; plasma cortisol level, 101.19 nmol/L; and thyroid-stimulating hormone, 22.251 mIU/L. The coagulation test results were as follows: prothrombin time, 1.87 seconds; activated partial thromboplastin time, 67.8 seconds; thrombin time, 25.8 seconds; fibrinogen level, 1.52 g/L; and D-dimer level, 1.29 mg/L. The hearing screening results were normal in both ears. There were no abnormalities in the liver, gallbladder, spleen, pancreas + urinary tract, brain, and thyroid ultrasound. The eye ultrasonography results were as follows: left axis, 17.0 mm; right axis, 16.1 mm. The left eyeball was slightly irregular. The cardiac ultrasonography results were as follows: secondary atrial septal defect (6 mm) and patent ductus arteriosus (2 mm). Computed tomography scan + three-dimensional reconstruction of the chest showed parenchymal infiltration between the lungs (pneumonia with local consolidation and absence of airway abnormalities). The patients received the following post-admission treatment: electrocardiogram oxygen monitoring, high-flow oxygen inhalation (5 L/min, 30%), and antibiotic treatment. Due to the unique appearance and multiple malformations of the child, 7 days after birth, the peripheral blood samples of the child and his parents were collected for genetic testing. There were gene returns at 35 days of age.

### 2.4. Gene detection method

This method uses genomic deoxyribonucleic acid (DNA) from the participant blood as the detection material, the DNA is interrupted, and the library is prepared. Then, the DNA of the target gene exon and near shear region is captured and enriched by Roche KAPA HyperExome chip. Finally, the MGISEQ-2000 sequencing platform is used for variant detection. In terms of the quality control index of sequencing data, the average sequencing depth of the target region is 180X, and the proportion of sites with a target region average depth of > 20x is > 95%. The data analysis results were as follows: The sequenced fragments were aligned to the UCSC hg19 human reference genome by Burrows–Wheeler Alignment tool to remove duplicates. Single nucleotide variant, insertion and deletion, and genotype tests were performed using Genome Analysis Toolkit. The copy number variation detection at the exon level was performed using ExomeDepth.

### 2.5. Genetic test results

**Figure 2.**
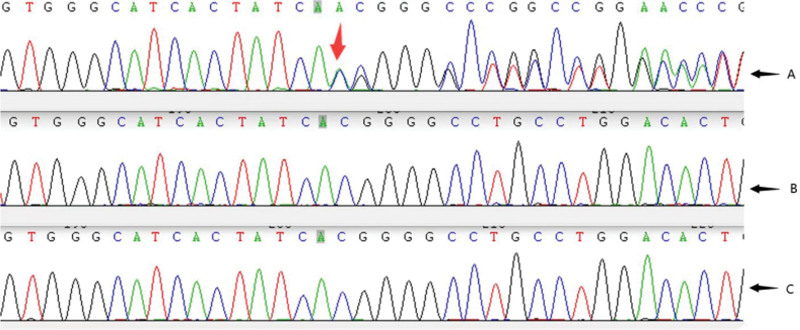
(A) TGenetic sequencing results of the child: the red arrow indicates that the child has a heterozygotic frameshift mutation of c.755dupA; (B) father gene sequencing results; (C) genetic sequencing results of the mother of the child.

A pathogenic mutation NM_003482.3:c.755dupA(p.His252Glnfs*21) was detected on the KMT2D gene associated with KS type 1, which was partially related to the phenotype of the subject. Both parents were wild type and KMT2D was detected NM_ 003482.3:c.755dupA (P.IS252GLNFS *21) changes (Fig. 2). This variant has not been reported (Table 1). According to the American College of Medical Genetics and Genomics guidelines, the variant is a pathogenic variant, and the variants are PVS 1 + PS2 + PM2. PVS 1 is characterized by a disease pathogenic mechanism that is loss of function. The detected variant is not functional (nonsense mutation, frameshift mutation, classical ± 1 or 2 splicing mutation, start codon variant, and single or multiple exon deletion). PS2 is characterized by a novel variant in a patient without a significant family history (validated by both parents). PM2 is characterized by variants not found in the normal control population in the Exome Sequencing Project, Thousand Person, or exome Aggregation Consortium databases.

**Table 1:** Genetic test results.

Gene	NM number	Gene subregion	Nucleotide alterations	Amino acid changes	Chromosomal position	Homozygous/heterozygous
KMT2D	NM_003482.3	EX6/CDS6	c.755dupA	p.His252Glnfs*21	Chr12:49447342–49447343	Heterozygosis

### 2.6. Diagnosis

The patient was diagnosed with heterozygous mutation of the NM_003482.3:c.755dupA(p.His252Glnfs*21) in *KMT2D*.

### 2.7. Treatment

There was no specific treatment for the disease, mainly the systematic follow-up of growth and development indicators, and symptomatic treatment for the clinical manifestations at different periods. By assessing the pathogenic genes causing KS, targeted therapy could be considered.

## 3. Discussion

KS is a rare genetic syndrome caused by mutations in X-linked histone H3 lysine 4 methylase (*KMT2D*) or H3 lysine 27 demethylase. In 2010, Ng et al performed exome sequencing of 10 patients with clinically diagnosed KS based on a specific scoring system, and confirmed the *KMT2D* variant as the causative gene for KS.^[6]^ *KMT2D* mutations cause disruption of histone methylation associated with gene expression, which affects normal growth and development. The facial features of patients with *KMT2D* truncation mutations are similar to those of KS. During infancy, the KMT 2 D mutation group had higher rates of experiencing clinical signs, such as hypotonia and facial deformity, than the KDM 6 A mutation group. Further, the *KMT2D* mutation group had a higher incidence of congenital heart disease than the KDM 6 A mutation group.^[7]^ The *KMT2D*is also closely related to the development of the nervous system, which is involved in regulating adhesion-related cytoskeletal events and promoting neuronal differentiation. *KMT2D* mutation can have neurostructural abnormalities, such as prolonged myelination in the brain white matter.^[8,9]^ The *KMT2D* can regulate cell development, metabolism, cell differentiation, and tumor suppression, leading to different dysmorphic disorders.^[10]^ Moreover, it has become one of the most commonly mutated genes^[11]^ in several types cancers, such as gastric cancer, lymphoma, and medulloblastoma.

In 2012, Miyake et al evaluated 3 patients with lysine K-specific demethylase 6A (*KDM6A*) gene deletion on the X chromosome.^[12]^ The *KDM6A* (OMIM * 300128) gene was the second causal gene in patients with KS, encoding a specific H3 lysine that interacts with *KMT2D. KDM6A* is located on Xp 11.3, and it encodes UTX.^[13]^ Previous studies have reported overlapping chromosome X microdeletions containing KDM 6A.^[14–16]^ Bank et al reported 7 patients with *KDM6A* mutations, causing type 2 KS, and they identified a germline splice site and missense mutation^[17]^ in *KDM6A*. Exon deletion of *KDM6A* is responsible for KS.

To date, most patients with KS (55%–90%) have *KMT2D* causal gene mutations. More than 50 mutations at different sites, including frameshift, nonsense and missense, and splice site mutations, have been identified. Truncated mutations were distributed throughout the coding region. Meanwhile, nonprotein truncated *KMT2D* mutations were mainly distributed around the functional domains. Less than 5% of KS cases are caused by mutations in *KDM6A*. Furthermore, patients without *KMT2D* or *KDM6A* mutations are commonly known as KS-like syndrome, with an incidence of approximately 30% of KS cases. *HNRNPK, RAPIA*, and *RAPIB* also cause pathogenicity.^[4]^ Previous studies have revealed that^[18,19]^ mutations in genes other than KMT2D or KDM6A should be considered in the routine diagnosis of mental retardation, evident facial deformity, and abnormal bone and connective tissue. The main clinical manifestations of KS are unique facial features, mental and growth retardation, hypotonia, feeding difficulties, skeletal abnormalities, immune dysfunction, endocrine abnormalities, congenital heart disease, and kidney and palate malformation. KMS 1 is more likely a characteristic than KMS 1 and KMS 2. The current diagnosis relies mainly on the identification of infantile hypotonia, developmental delay, or intellectual disability, combined with typical dysmorphic features and KMT2D or KDM6A mutations based on genetic testing. To promote the clinicians’ awareness of KS, the international consensus established the diagnostic criteria in 2019. The International Panel recommends that the diagnosis of KS^[20]^ can be made at any age inpatients with a history of infantile hypotonia, developmental delay and/or intellectual disability, and one or both of the following main criteria:

1. A pathogenic or likely pathogenic variant in either the *KMT2D* or *KDM6A*.
2. Typical dysmorphic features, which include long eyelid fissure (measurements greater than or more than the average age),and 2 or more of the following: arched and wide eyebrows and nicked and sparse lateral third of the lower eyelid, short columella and sunken nose tip, large, prominent, or pointed ears, and long-lasting fingertip pad.

The clinical diagnosis of KS often requires long-term monitoring because clinical characteristic malformations and other essential features are often detected a few years after birth. Prenatal, neonatal periods are rarely found and have no clear family history. Facial dysmorphism may raise suspicion of KS, and a final definitive diagnosis requires genetic testing. According to previous reports, the diagnosis of KS is made at ages 3 months to 22 years old. However, the average age of diagnosis is approximately 6.4 years old, and the first case of KS in a 4-year-old child in China was reported in 2010.^[21,22]^ Thus, the number of related case reports in our country is still low, and our understanding on KS must be further improved. The earliest diagnostic time was at 40 days old.^[23]^ The condition was diagnosed at the neonatal period. That is, 2 hours after birth, she was admitted to the neonatal department due to hypoglycemia and shortness of breath. Physical examination found that the child face was unique (long eye crack, small eyeball in the left eye, short nose column, large ears, webbed neck, and pass-through palm), as shown in Figure 1. Subsequent inpatient examinations revealed the presence of multiple malformations, including atrial septal defect (6 mm), patent ductus arteriosus (2 mm), hidden penis, abnormal palmprint, hypoglycemia, and high thyroid-stimulating hormone levels; therefore, genetic test was performed at 7 and 35 days afterbirth. The results of the test confirmed that the child had KS, which was caused by heterozygous mutation in KMT2D;NM_003482.3:c.755dupA(p.His252Glnfs*21). Therefore, it is extremely important to identify the diagnosis based on genetic test results and multiple malformations in the neonatal period. Niikawa et al found that based on the physical features of KS on early assessments, children with KS had normal birth weight and body length and postnatal growth retardation^.[24]^ In the current case of KS, weight and length growth did not lag behind 42 days after diagnosis, and further follow-up on growth and development was required. According to current reports, the common developmental abnormalities include poor physical growth, intellectual impairment, cardiac, gastrointestinal, and renal abnormalities, and behavioral issues including autistic features.^[25]^ In this case, typical facial features (such as long eye fissure, nicked and sparse lateral third of the lower eyelid, short nasal column, large ears, and abnormal development of the heart and external genitalia, and endocrine metabolism abnormalities) can be observed. This finding is consistent with that of previous reports. Due to the short follow-up time, this child should be monitored subsequently. The involvement of *KMT2D* and *KDM6A* mutations in insulin metabolism has not been identified. However, hyperinsulinemic hypoglycemia is observed in 0.3% to 4% of patients with KS patients, commonly occurring^[26]^ on the first day of life. In our case, hypoglycemia was the primary symptom of hospitalization. Thus, it was diagnosed in the neonatal period. After admission, hypoglycemia was corrected with glucose supplementation, and the subsequent review was normal. Up to 40% of patients with KS develop urinary abnormalities, with about half presenting with renal malformations and half with urinary tract abnormalities. The etiology of urinary abnormalities in KS is believed to be caused by the role of *KMT2D* epigenetics in the regulation of renal development. *KMT2D* dysfunction may eventually lead to renal dysplasia. However, the specific role of *KMT2D* in kidney development has not been determined.^[27]^ In our case, the patient presented with occult penis. Some clinical reports revealed immunodeficiency or autoimmune surface diseases, such as immune thrombocytopenic purpura,^[28]^ systemic lupus erythematosus,^[29]^ and hypogammaglobulinemia,^[30]^ in patients with KS. However, the prevalence of KS differs according to gene type^.[31]^ At present, the treatment of patients with KS in China and other countries is mainly symptomatic treatment. Management with increased nutrition and feeding volume and positioning is adopted for children with gastroesophageal reflux. Moreover, gastrostomy catheterization is feasible for children with extreme feeding difficulties. Based on the growth and intelligence assessment, early surgery for deformity correction, such as the treatment of congenital heart disease,^[32]^ glaucoma,^[33]^ and cleft lip and palate,^[34,35]^ should be considered in the early disease stage in children with intelligence training. Meanwhile, children with autism who have evident cognitive impairment should undergo psychological education test and avail special education services offered by the pediatrician or psychiatry expert professionals.^[36]^ The surviving children are followed up once a year. That is, their height, body mass, and head circumference are evaluated.^[37]^ Children with growth retardation can receive growth hormone therapy.^[38]^ The development and validation of the KS gene in animal models can improve KS phenotypic traits from molecular mechanisms in diseases caused by *KMT2D* mutations via a possible therapeutic approach.^[15]^ The pathogenesis of KS is associated with a disorder between chromatin opening and closure,^[18]^ and drugs inducing chromatin opening may be used for the treatment of KS in the future. Both *KMT2D* and *KDM6A* act as epigenetic regulators via histone modifications,^[39]^ and small-molecule inhibitors of histone deacetylases can be used for the treatment of intellectual disability and can reduce the risk of cancer in KS.^[40]^ We report a case of neonatal KS caused by heterozygous gene mutation in KMT2D;NM_003482.3:c.755dupA(p.His252Glnfs*21). Caused by a genetic mutation in a newborn. Because the clinical phenotype in the neonatal period is not obvious, few cases have been reported. Our report provides reference data for clinical diagnosis and genetic counseling of the disease. The limitation lies in the lack of follow-up and treatment of growth and development. The molecular etiological mechanism of the disease needs further investigation with more cases. In conclusion, clinicians should improve their knowledge about KS, early diagnosis, systematic follow-up, and symptomatic treatment to improve patient prognosis. KS has a strong clinical and biological heterogeneity. Its features include unique facial features, intellectual disability, developmental delay, prominent toe pads, skeletal and visceral malformations, endocrine disorders, and autoimmune diseases. Approximately 70% of patients with KS have pathogenic *KMT2D* or *KDM6A* variants. At present, KS is mainly managed with symptomatic treatment for various developmental malformations, and the study of gene function maybe a therapeutic method for future targeted therapy of molecular mechanisms.

## Acknowledgments

We would like to thank the parents for providing us patient information and entrusting us with their data. We are grateful for their contribution to the progress of research on Kabuki syndrome. We are also grateful to our colleagues for their cooperation in conducting this work and treating this patient. We would also like to thank English Pavilion for providing English editing service.

## Author contributions

**Conceptualization:** Zhang Li, Zou Ning.

**Writing – original draft:** Zhang Li.

**Writing – review & editing:** Zou Ning.
